# The impact of smoking on male lower urinary tract symptoms (LUTS)

**DOI:** 10.1038/s41598-020-77223-7

**Published:** 2020-11-19

**Authors:** Takashi Kawahara, Hiroki Ito, Hiroji Uemura

**Affiliations:** 1 Departments of Urology and Renal Transplantation, Yokohama City University Medical Center, 4-57, Urafune-cho, Minami-ku, Yokohama, 2320024 Japan; 2 Department of Urology, Yokohama City University, Graduate School of Medicine, Yokohama, Japan

**Keywords:** Urology, Bladder

## Abstract

Lower urinary tract symptoms (LUTS) are substantially prevalent and increase with age. Research on smoking as a risk factor for LUTS has been inconclusive. The present study examined the association between smoking habits and male LUTS in a population-based study using a web-based questionnaire. We firstly screened a total of 10,000 male participants who were selected according to the age distribution in the Japanese population in government data, in order to check smoking habits. We then performed a web-based survey to further investigate factors associated with LUTS, using the Overactive Bladder Symptom Score (OABSS), International Consultation on Incontinence Questionnaire-Short Form (ICIQ-SF), and International Prostate Symptom Score (IPSS) questionnaire. Finally, 9042 participants (non-smokers, n = 3545; ex-smokers, n = 3060; and current-smokers, n = 2437) completed the full continence survey. Current-smokers (2.54 ± 2.73, 1.98 ± 3.57, 5.75 ± 7.02) and ex-smokers (2.80 ± 2.52, 1.81 ± 3.10, 6.58 ± 6.96) showed significantly higher OABSS total, ICIQ-SF total, and IPSS total scores than non-smokers (1.98 ± 2.40, 1.35 ± 2.90, 4.23 + -/6.33) (p: < 0.0001, < 0.0001, < 0.0001, respectively). In comparison to non-smokers, the prevalence of risk ratio for day-time frequency, nocturia, urgency urinary incontinence (UUI), OAB, and IPSS ≥ 8 were 1.2 1.2 1.4 1.5 1.5, respectively, in current-smokers and 1.3, 1.5, 1.5, 4.5, 1.8 in ex-smokers. The relative risk of OAB, nocturia, UUI, and IPSS ≥ 8 in ex- and current-smokers in comparison to non-smokers was high in the young age groups in comparison to the elderly groups. Current-smokers and ex-smokers showed a higher prevalence of male LUTS. This phenomenon was highly observed in relatively young age groups.

## Introduction

Lower urinary tract symptoms (LUTS), which consist of storage, voiding, and postmicturition symptoms and decreased health-related quality of life, were first introduced in 1997^1^. LUTS were also detected by individuals with pathologies affecting the urinary tract, including detrusor overactivity, sphincteric weakness, sensory bladder disorders, and benign prostatic hyperplasia (BPH)^2^.

Previous studies have reported that LUTS, including OAB, are multi-factorial in both men and women^3,4^. The most important factor is aging. Diabetes mellitus, hypertension, and psychological factor were reported to the risk factor for LUTS, and these risk factors are correlated with aging^5^. Especially for males, BPH has a negative impact for elderly. On the other hand, not a few patients were suffered from LUTS and lowers quality of day life.

We previously reported the impact of smoking for female LUTS and both current- and ex- smoking had a worse risk factor for female LUTS especially for young generation^6^. Some studies have indicated that smoking is a risk factor for LUTS^7,8^. However, research on smoking as a risk factor for LUTS has been inconclusive, with some studies finding that current-smokers are at greater risk of developing LUTS than non-smokers^9,10^ and others reporting no association or a possible inverse relationship between current-smoking and LUTS^11,12^. We examined the association between smoking habits and male LUTS in a population-based study using a web-based questionnaire.

## Materials and methods

### Screening survey to determine smoking habits

A total of 10,000 male individuals were recruited from monitoring lists of 4.5 million panels (approximately 45,000 individuals) at a web-based Internet survey company (Freeasy; iBRIDGE Company, Tokyo, JAPAN). Initially 10,000 individuals were selected according to the age and sex distribution of the Japanese population based on government data, and were asked about their smoking habits. These 10,000 individuals were firstly recruited by e-mail from 4.5 million panels according to age distribution and after reached to the number of participants in each generation no more individuals were recruited. According to the research company usually approximately 60% of participants answered by smartphone or tablet and the other 40% participants answered by PC. The respondents were classified into three categories according to smoking habits: (1) non-smokers, (2) ex-smokers, and (3) current smokers. For ex-smokers, we asked about the duration of smoking cessation and for current-smokers, we asked about the duration of smoking and the number cigarettes smoked per day. We then asked these participants about their detailed urinary symptoms. The following information, obtained on registration, was also included in the analysis: age, marital status, number of children, household income, working status, and prefecture of residence. Informed consent was obtained all participants and the study received approval from the Institutional Review Board (IRB) of Yokohama City University Medical Center (Yokohama, Japan) (IRB No. B200500015). This study was carried out in compliance with the Declaration of Helsinki.

### Questionnaire

We evaluated urinary symptoms using the Japanese version of the validated Overactive Bladder Symptom Score (OABSS), International Consultation on Incontinence Questionnaire-Short Form (ICIQ-SF), the International Prostate Symptom Score (IPSS), and the Quality of Life Score (QOL score)^13,14^.

The OABSS, originally developed in Japan, is a 4-item questionnaire that expresses OAB symptoms on a single scale^13^. The OABSS question items address the following individual symptoms: daytime frequency, nocturia, urgency, and urgency incontinence. Gotoh et al. reported that the OABSS was useful for assessing the effects of treatment on OAB symptoms and was responsive to treatment-related changes^15^. The OABSS score was defined as the sum of the total OABSS scores and OAB was defined the presence of both a total score of ≥ 3 and an OABSS Q3 score of ≥ 2. The OABSS defined urgency urinary incontinence (UUI) as an OABSS Q4 score of ≥ 1. Day-time frequency was defined as an OABSS Q1 score of ≥ 1. Nocturia was defined as an OABSS Q2 score of ≥ 2.

The ICIQ-SF was developed to screen for incontinence, obtain a brief yet comprehensive summary of the level, impact, and perceived causes of symptoms of incontinence, and facilitate patient-clinician discussions^16^. The ICIQ-SF score was calculated as the sum of the Q1, Q2, and Q3 scores. UUI (ICIQ-SF definition) was defined by a positive response to “leaks occur before you can get to the toilet”. Stress urinary incontinence (SUI) was defined by a positive response to at least one of the following: “leaks occur when you cough or sneeze” and “leaks occur when you are physically active/exercising”. Mixed urinary incontinence (MUI) was defined as both UUI (ICIQ-SF definition) and SUI. Post-micturition dribble (PMD) was defined as a positive response to “leaks occur when you have finished urinating and are dressed”.

The first version of the IPSS was created in 1992 by the American Urological Association. The IPSS consisted of seven questions relating to symptoms experienced in the last month, including a feeling of incomplete bladder emptying, frequency of urination, intermittency of urine stream, urgency of urination, weak stream, straining and waking at night to urinate^14^. The current study used the Japanese language validated IPSS and QOL score to assess storage and voiding symptoms^17^. In the IPSS, nocturia was defined by an IPSS Q7 score of ≥ 1. The IPSS storage score used the sum of IPSS Q2, 4, and 7 and the IPSS voiding score used the sum of Q3, 5, and 6. Individuals with a total IPSS score of ≥ 8 were classified as moderately symptomatic.

### Statistical analyses

The participants’ characteristics and scores were analyzed by the Mann–Whitney *U* test and correlation was analyzed by Spearman correlation coefficient test using the Graph Pad Prism software program (Graph Pad Software ver7.05, La Jolla, CA, USA). P values of < 0.05 were considered to indicate statistical significance.

## Results

**Figure 1:**
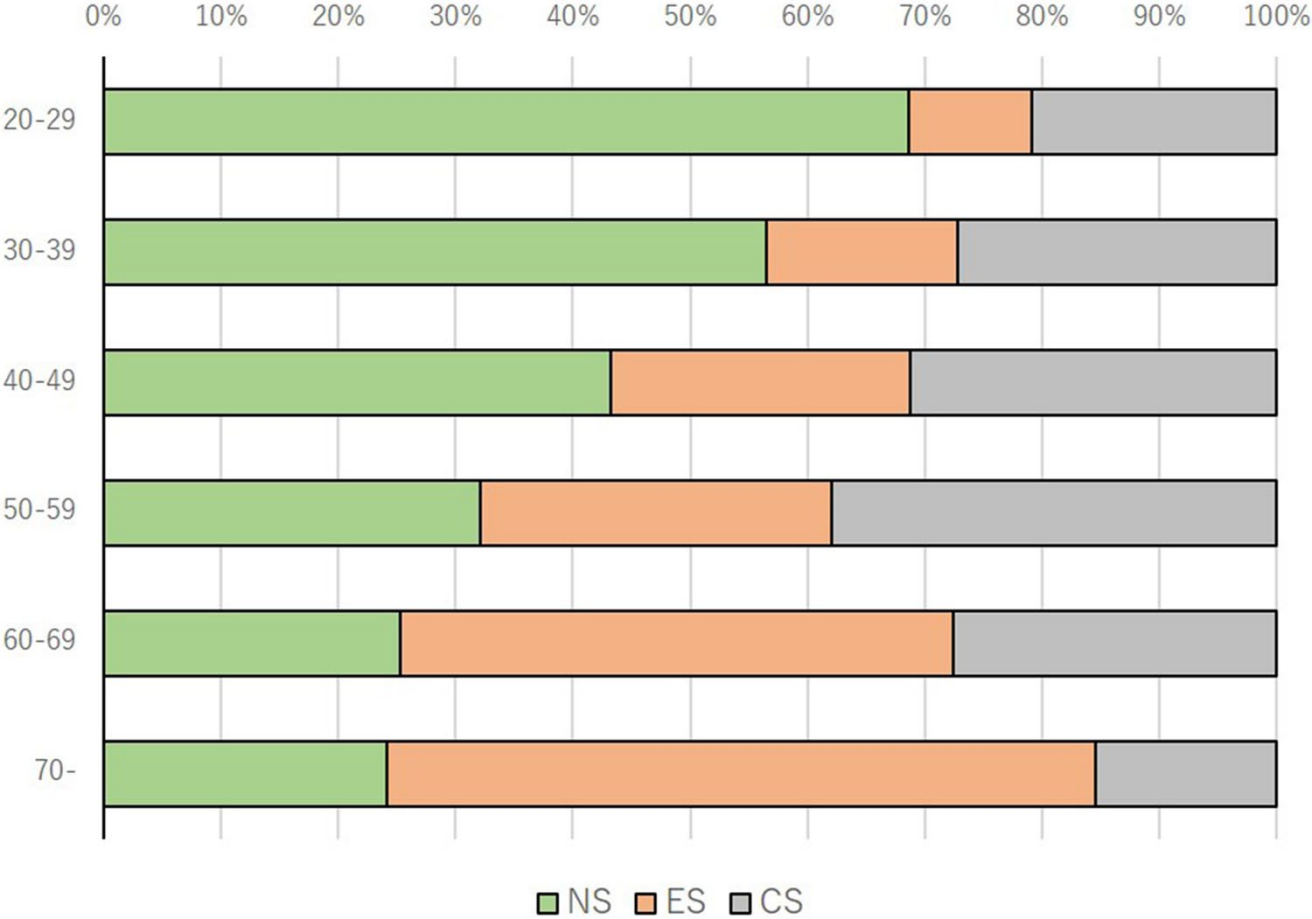
Study design and the number of participants.

**Figure 2:**
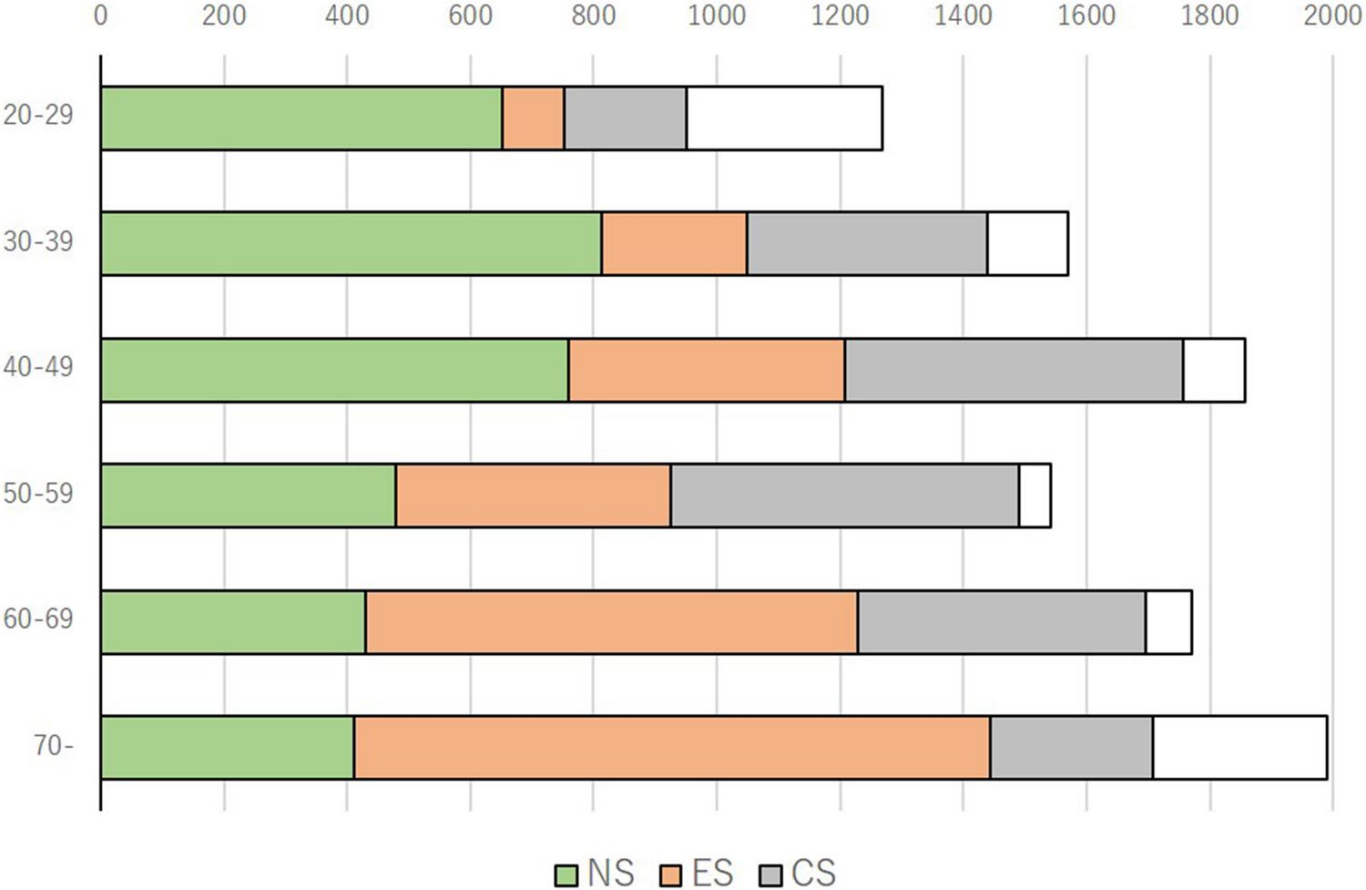
Number of targeted screening participants. Blank bar is the differences between targeted number of participants and the actual number pf participants who answered the questionnaire in each generation group.

We first screened 10,000 men which the number of males in each generation was adjusted to the age distribution according to the Japanese population based on government data, in order to check the smoking status^18^. In this screening, 3545 (39.2%) respondents were non-smokers, 3060 (33.8%) were ex-smokers, and 2437 (27.0) were current-smokers (Fig. 1). The age distribution and the percentage of non-, ex-, and current-smokers are shown in Fig. 2 and Supplementary Fig. S1. The percentage of non-smokers decreased in correlation with age, and the percentage of ex-smokers increased in correlation with age. The percentage of current-smokers peaked in the 50–59 years age group. The prefecture of residence is shown in Supplementary Table S1. The participants’ background characteristics, including age, marital status, number of children, household income, working status, and prefecture of residence, are listed in Table 1. For the screening survey, we developed web-based questionnaire and reached to short answering time. The mean time taken to complete the smoking status questionnaire was 49 s. For the continence survey, mean time taken to complete the questionnaire was 2 min and 46 s.

**Table 1:** Background characteristics.

	All	Non-smoking	Ex-smoking	Current-smoking
	n = 9042	n = 3545	n = 3060	n = 2437
**Age (years)**				
20–29	951 (10.5%)	653 (18.4%)	99 (3.2%)	199 (8.2%)
30–39	1440 (15.9%)	813 (22.9%)	236 (7.7%)	391 (16.0%)
40–49	1756 (19.4%)	759 (21.4%)	448 (14.6%)	549 (22.5%)
50–59	1491 (16.5%)	479 (13.5%)	446 (14.6%)	566 (23.2%)
60–69	1697 (18.8%)	429 (12.1%)	799 (26.1%)	469 (19.2%)
70 or more	1707 (18.9%)	412 (11.6%)	1032 (33.7%)	263 (10.8%)
**Status of marriage**				
Unmarried	3586 (39.7%)	1940 (54.7%)	694 (22.7%)	952 (39.1%)
Married	5456 (60.3%)	1605 (45.3%)	2366 (77.3%)	1485 (60.9%)
**Working pattern**				
Employee	3587 (39.7%)	1497 (42.2%)	938 (42.2%)	1152 (47.3%)
Civil servant	319 (3.5%)	166 (4.7%)	70 (4.7%)	83 (3.4%)
Free-lance	229 (2.5%)	82 (2.3%)	72 (2.3%)	75 (3.1%)
Housewife	30 (0.3%)	6 (0.2%)	16 (0.2%)	8 (0.3%)
Manager	225 (2.5%)	60 (1.7%)	85 (1.7%)	80 (3.3%)
Medical worker	104 (1.2%)	48 (1.4%)	33 (1.4%)	23 (0.9%)
Part-time job	631 (7.0%)	303 (8.5%)	183 (8.5%)	145 (5.9%)
Self owned work	596 (6.6%)	167 (4.7%)	245 (4.7%)	184 (7.6%)
Student	186 (2.1%)	149 (4.2%)	11 (4.2%)	26 (1.1%)
Temporary worker	552 (6.1%)	227 (6.4%)	170 (6.4%)	155 (6.4%)
No occupation	2332 (25.8%)	727 (20.5%)	1158 (20.5%)	447 (18.3%)
Others	251 (2.8%)	113 (3.2%)	79 (3.2%)	59 (2.4%)
**Annual household income**				
-2,000,000 JPY	1339 (14.8%)	574 (16.2%)	434 (14.2%)	331 (13.6%)
2,000,000–4,000,000 JPY	2423 (26.8%)	936 (26.4%)	924 (30.2%)	563 (23.1%)
4,000,000–6,000,000 JPY	2138 (23.6%)	830 (23.4%)	736 (21.1%)	572 (23.5%)
6,000,000–8,000,000 JPY	1311 (14.5%)	485 (13.7%)	435 (14.2%)	391 (16.0%)
8,000,000–10,000,000JPY	815 (9.0%)	309 (8.7%)	233 (7.6%)	273 (11.2%)
10,000,000 J-PY	1016 (11.2%)	411 (11.6%)	298 (9.7%)	307 (12.6%)
**Residency**				
Own house (detached house)	4988 (55.2%)	1825 (51.5%)	1885 (61.6%)	1278 (52.4%)
Own house (condominium)	1391 (15.4%)	506 (14.3%)	511 (16.7%)	374 (15.3%)
Lease (apartment)	903 (10.0%)	419 (11.8%)	214 (7.0%)	270 (11.1%)
Lease (condominium)	1057 (11.7%)	459 (12.9%)	256 (8.4%)	342 (14.0%)
Lease (detached house)	271 (3.0%)	113 (3.2%)	94 (3.1%)	64 (2.6%)
Company house	103 (1.1%)	50 (1.4%)	24 (0.8%)	29 (1.2%)
Dormitory	65 (0.7%)	43 (1.2%)	9 (0.3%)	13 (0.5%)
Others	264 (2.9%)	130 (3.7%)	67 (2.2%)	67 (2.7%)
**Presence of child**				
Yes	4334 (47.9%)	2251 (63.5%)	965 (31.5%)	1118 (45.9%)
No	4708 (52.1%)	1294 (36.5%)	2095 (68.5%)	1319 (54.1%)

**Figure 3:**
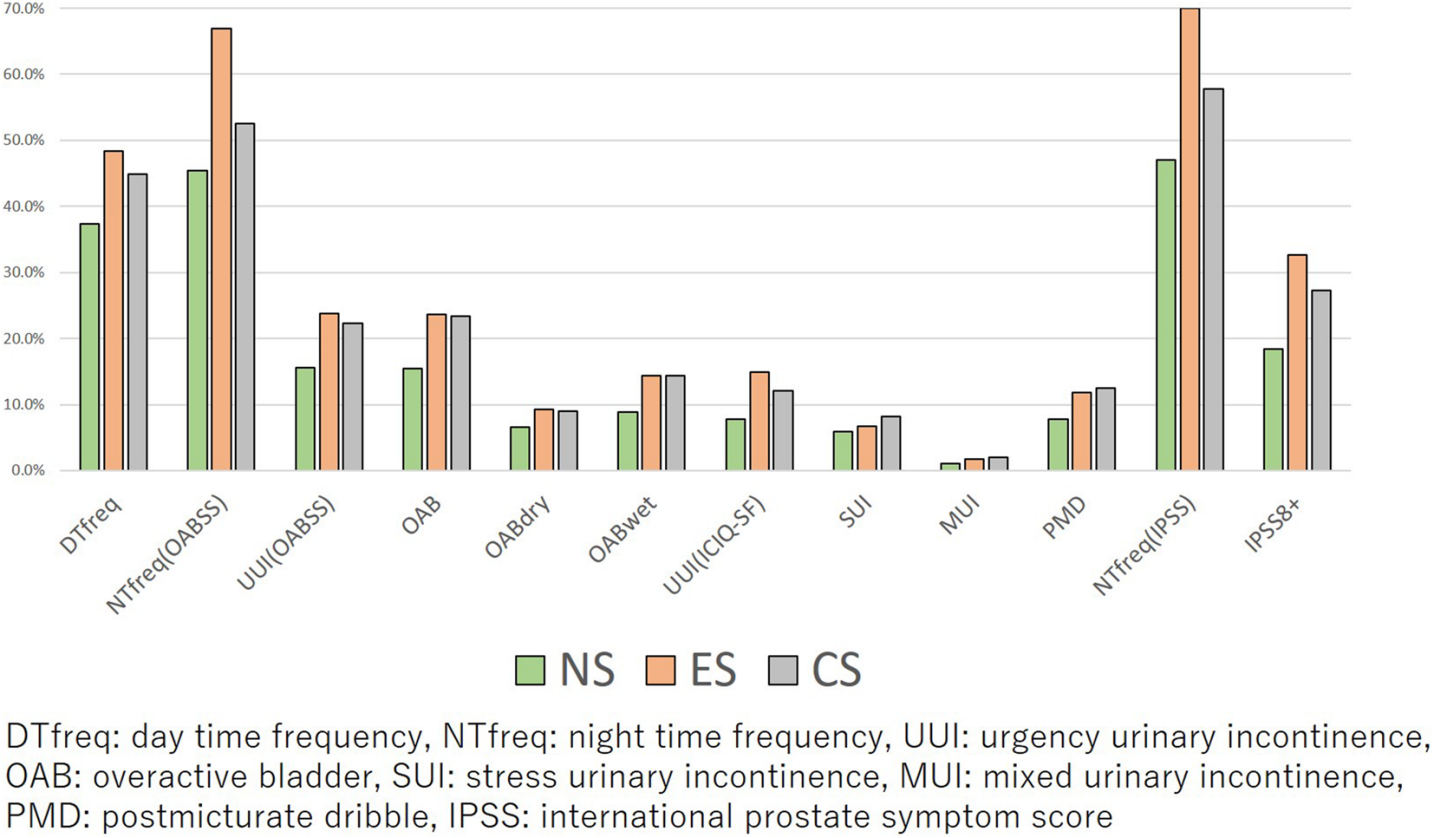
The prevalence of day-time frequency, nocturia (OABSS), UUI (OABSS definition), OAB, OAB dry, OAB wet, UUI (ICIQ-SF), SUI, MUI, PMD, nocturia (IPSS definition), and IPSS ≥ 8 in non-, ex-, and current-smokers.

The prevalence of day-time frequency, nocturia (OABSS definition), UUI, OAB (OABSS definition), OAB dry, OAB wet, UUI (ICIQ-SF definition), SUI, MUI, PMD, nocturia (IPSS definition), and IPSS ≥ 8 for each smoking status is shown in Fig. 3. Ex- and current-smokers showed a higher prevalence of all LUTS symptoms in comparison to non-smokers. Ex-smokers showed a higher prevalence of day-time frequency, nocturia, UUI (ICIQ-SF), and IPSS ≥ 8 than current-smokers. Current-smokers showed a higher prevalence of SUI, MUI, and PMD than ex-smokers. For the other symptoms, the prevalence was almost the same in ex- and current-smokers (Supplementary Fig. S2).

For OABSS, all scores (Q1-4) and the total score were significantly higher in ex- and current-smokers than in non-smokers (p: < 0.0001, < 0.0001, < 0.0001, < 0.0001, < 0.0001 in non- vs. ex-smokers, < 0.0001, < 0.0001, < 0.0001, < 0.0001, < 0.0001 in non- vs. current-smokers respectively). And OABSS Q2 score and total score were significantly higher in ex-smokers than in current-smokers (p < 0.0001 and 0.0002, respectively). The other categories showed no significant differences between ex- and current-smokers (Table 2).

**Table 2:** OABSS, ICIQ-SF, and IPSS scores of non-smokers, ex-smokers, and current smokers.

Variables	Non-smoking	Ex-smoking	Current-smoking	p value		
	n = 3540	n = 3060	n = 2437	NS vs ES	NS vs CS	ES vs CS
**OABSS_Q1**						
Means ± SD	0.41 ± 0.56	0.53 ± 0.58	0.50 ± 0.59	< 0.0001	< 0.0001	0.0647
Participants scoring one or more (n, %)	1326, 37.4%	1480, 48.4%	1092, 44.8%			
**OABSS_Q2**						
Means ± SD	0.64 ± 0.83	1.00 ± 0.90	0.74 ± 0.86	< 0.0001	< 0.0001	< 0.0001
Participants scoring one or more (n, %)	1608, 45.4%	2049, 67.0%	1280, 52.5%			
**OABSS_Q3**						
Means ± SD	0.66 ± 1.03	0.89 ± 1.13	0.91 ± 1.17	< 0.0001	< 0.0001	0.5603
Participants scoring one or more (n, %)	1402, 39.5%	1523, 49.8%	1212, 49.7%			
**OABSS_Q4**						
Means ± SD	0.27 ± 0.77	0.39 ± 0.82	0.40 ± 0.90	< 0.0001	< 0.0001	0.6695
Participants scoring one or more (n, %)	550, 15.5%	885, 28.9%	545, 22.4%			
**OABSS total score**						
Means ± SD	1.98 ± 2.40	2.80 ± 2.52	2.54 ± 2.73	< 0.0001	< 0.0001	0.0002
Participants scoring one or more (n, %)	2413, 68.1%	2454, 83.2%	50, 75.9%			
**ICIQ-SF Q1**						
Means ± SD	0.28 ± 0.77	0.39 ± 0.82	0.40 ± 0.88	< 0.0001	< 0.0001	0.8832
Participants scoring one or more (n, %)	573, 16.2%	774, 25.3%	567, (23.3%)			
**ICIQ-SF Q2**						
Means ± SD	0.47 ± 1.02	0.64 ± 1.06	0.69 ± 1.17	< 0.0001	< 0.0001	0.0947
Participants scoring one or more (n, %)	719, 20.3%	885, 28.9%	711, 29.2%			
**ICIF-SQ Q3**						
Means ± SD	0.60 ± 1.52	0.78 ± 1.61	0.90 ± 1.89	< 0.0001	< 0.0001	0.0109
Participants scoring one or more (n, %)	747, 21.1%	909, 29.7%	714, 29.3%			
**ICIQ-SF total score**						
Means ± SD	1.35 ± 2.90	1.81 ± 3.10	1.98 ± 3.57	< 0.0001	< 0.0001	0.0533
Participants scoring one or more (n, %)	953, 26.9%	1094. 35.8%	858, 35.2%			
**IPSS Q1**						
Means ± SD	0.59 ± 1.12	0.80 ± 1/21	0.80 ± 1.21	< 0.0001	< 0.0001	0.9777
Participants scoring one or more (n, %)	1154, 32.6%	1382, 45.2%	1087, 44.6%			
**IPSS Q2**						
Means ± SD	0.81 ± 1.24	1.13 ± 1/38	1/03 ± 1.31	< 0.0001	< 0.0001	0.0040
Participants scoring one or more (n, %)	1544, 43.6%	1769, 57.8%	1301, 53.4%			
**IPSS Q3**						
Means ± SD	0.48 ± 1.13	0.79 ± 1.34	0.68 ± 1.26	< 0.0001	< 0.0001	0.0015
Participants scoring one or more (n, %)	777, 21.9%	1112, 36.3%	758, 31.1%			
**IPSS Q4**						
Means ± SD	0.40 ± 0.96	0.57 ± 1.09	0.54 ± 1.06	< 0.0001	< 0.0001	0.2688
Participants scoring one or more (n, %)	757, 21.4%	971, 31.7%	717, 29.4%			
**IPSS Q5**						
Means ± SD	0.62 ± 1.28	1.22 ± 1.64	0.91 ± 1.43	< 0.0001	< 0.0001	< 0.0001
Participants scoring one or more (n, %)	976, 27.5%	1531, 50.0%	997, 40.9%			
**IPSS Q6**						
Means ± SD	0.48 ± 1.15	0.80 ± 1.37	0.69 ± 1.30	< 0.0001	< 0.0001	0.0036
Participants scoring one or more (n, %)	761, 21.5%	1096, 35.8%	754, 30.9%			
**IPSS Q7**						
Means ± SD	0.84 ± 1.21	1.26 ± 1.24	1.09 ± 1.35	< 0.0001	< 0.0001	< 0.0001
Participants scoring one or more (n, %)	1669, 47.1%	2140, 66.9%	1407. 57.7%			
**IPSS storage**						
Means ± SD	2.05 ± 2.69	2.96 ± 2.87	2.66 ± 2.95	< 0.0001	< 0.0001	0.0002
Participants scoring one or more (n, %)	2186, 61.7%	2461, 80.4%	1764, 72.4%			
**IPSS voiding**						
Means ± SD	1.59 ± 3.23	2.81 ± 3.86	2.29 ± 3.58	< 0.0001	< 0.0001	< 0.0001
Participants scoring one or more (n, %)	1193, 33.7%	1682, 55.0%	1141, 46,8%			
**IPSS total**						
Means ± SD	4.23 ± 6.33	6.58 ± 6.96	5.75 ± 7.02	< 0.0001	< 0.0001	< 0.0001
Participants scoring one or more (n, %)	2308, 65.1%	2561, 83.7%	1861, 76.4%			
**QOL**						
Means ± SD	2.23 ± 1.58	2.69 ± 1.61	2.55 ± 1.64	< 0.0001	< 0.0001	0.0015
Participants scoring one or more (n, %)	2853, 80.5%	2688, 87.8%	2088, 85.7%			

*NS* non-smoking, *ES* ex-smoking, *CS* current-smoking.

For ICIQ-SF, Q1-Q3 and total score were also significantly higher in ex- and current- smokers than in non-smokers (p: < 0.0001, < 0.0001, < 0.0001, < 0.0001, < 0.0001 in non- vs. ex-smokers, < 0.0001, < 0.0001, < 0.0001, < 0.0001, < 0.0001 in non- vs. current-smokers respectively). In ICIQ-SF Q3, the score of current-smokers was higher than that of ex-smokers (0.90 ± 1.89 vs. 0.78 ± 1.61, p: 0.0109) (Table 2).

For IPSS and QOL, all (Q1–7), storage, voiding, total, and QOL scores were higher in ex- and current-smokers than in non-smokers (all p-values < 0.0001) (Table 2). When ex- and current-smokers were compared, all of the above categories, with the exception of the IPSS Q1 and Q4 scores, were significantly higher in ex-smokers than in current-smokers. The IPSS Q1 and Q4 scores did not differ between ex- and current-smokers.

**Figure 4:**
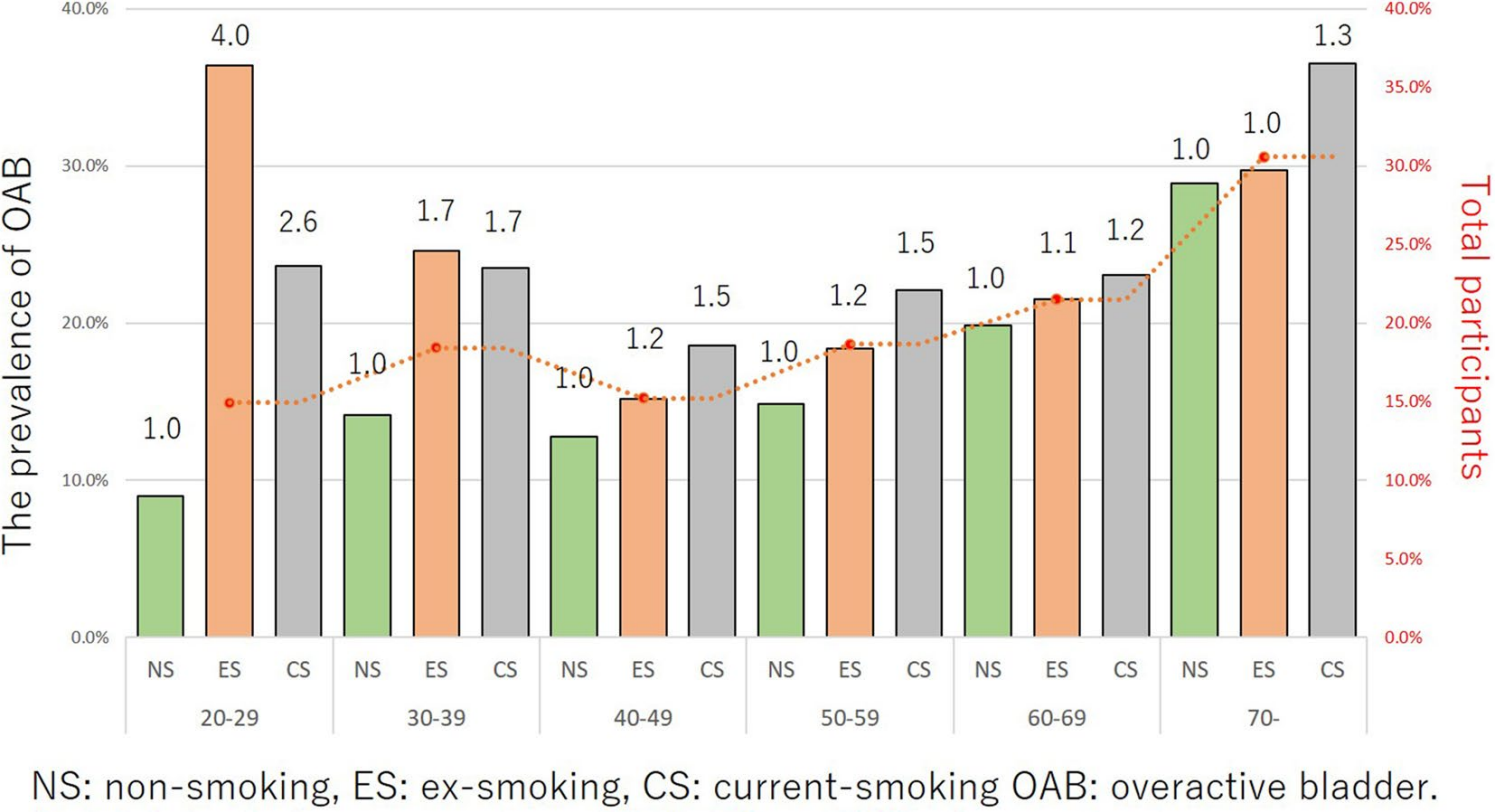
The prevalence of OAB in each age group among the different smoking habit groups. Red dot line is the mean values of NS, ES, and CS in these age group.

**Figure 5:**
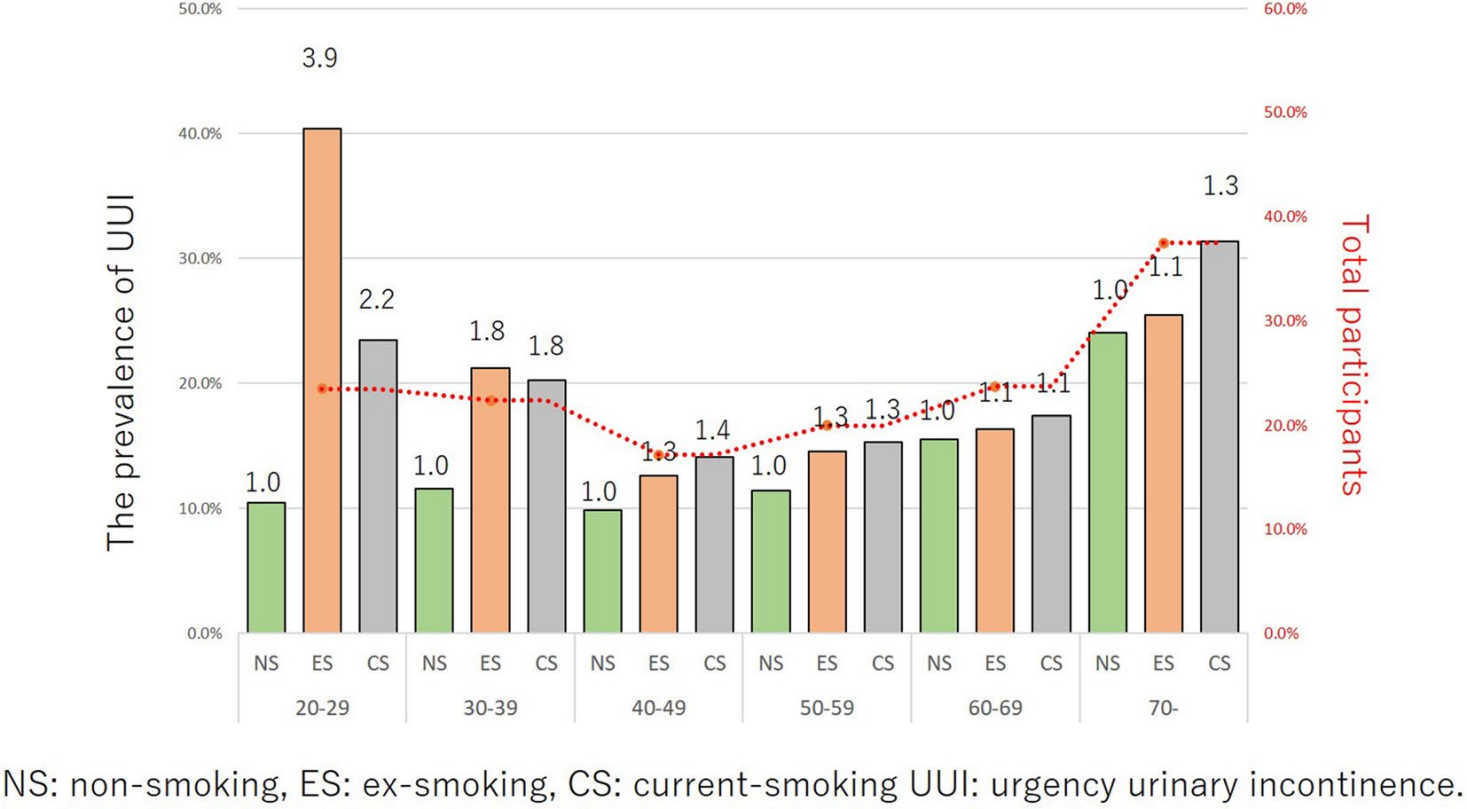
The prevalence of UUI in each age group among the different smoking habit groups. Red dot line is the mean values of NS, ES, and CS in these age group.

**Figure 6:**
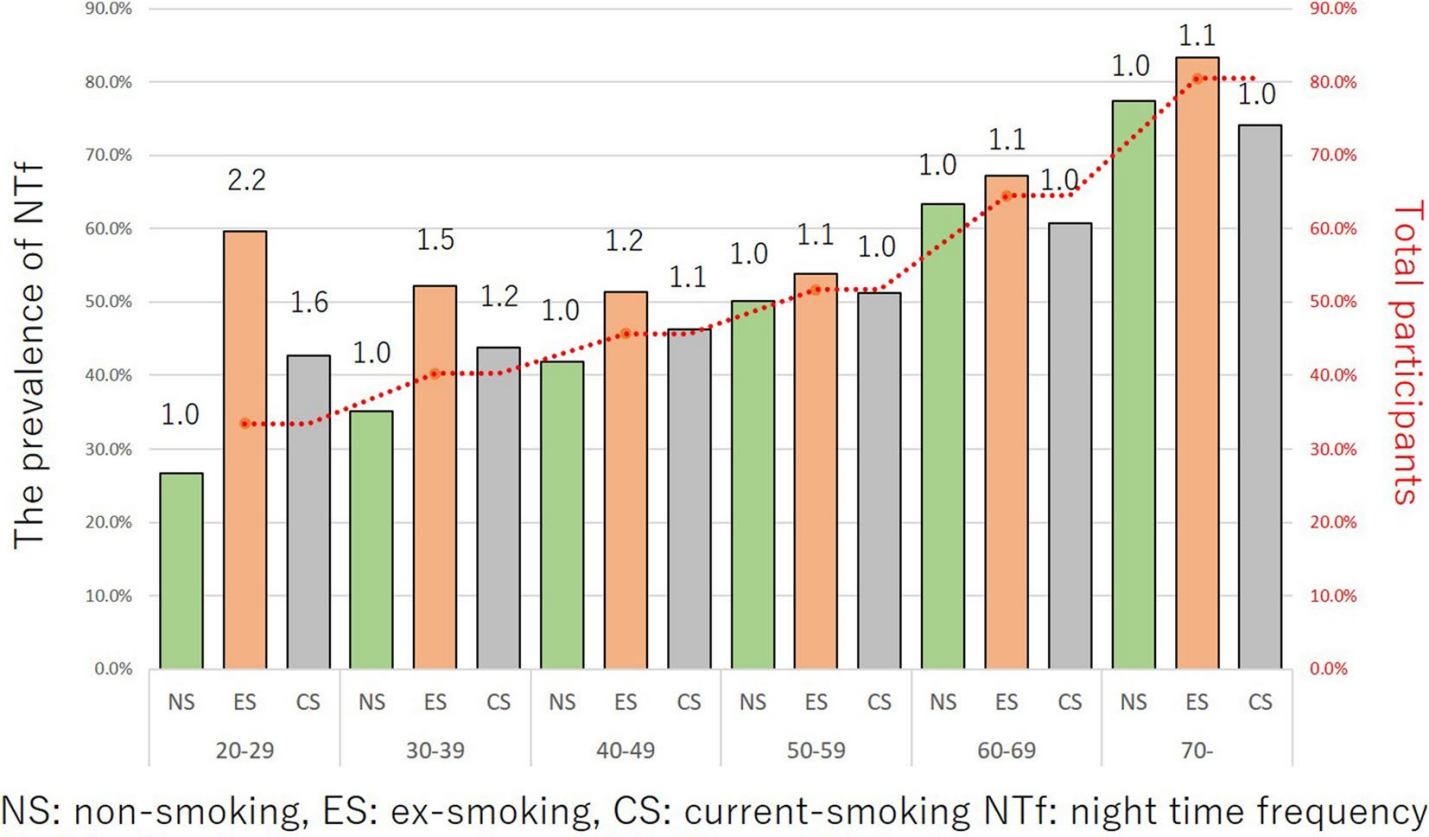
The prevalence of nocturia in each age group among the different smoking habit groups. Red dot line is the mean values of NS, ES, and CS in these age group.

**Figure 7:**
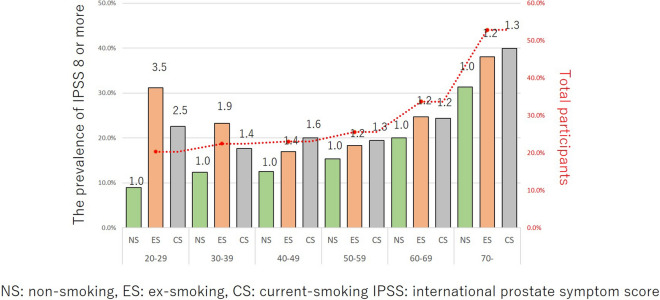
The prevalence of IPSS ≥ in each age group among the different smoking habit groups. Red dot line is the mean values of NS, ES, and CS in these age group.

The correlation between the smoking status and OAB, UUI, nocturia, and IPSS ≥ 8 in each age group are shown in Figs. 4, 5, 6 and 7. The prevalence of OAB, nocturia, and IPSS ≥ 8 were positively correlated with age, but not positively correlated with UUI due to the higher incidence of young age group (OAB: r = 0.8408, p = 0.036, UUI: r = 0.5801, p = 0.2274, nocturia: r = 0.9729, p = 0.0011, and IPSS ≥ 8: r = 0.8663, p = 0.0256). Totally, 20.3% were detected as OAB. The prevalence of OAB, nocturia, and IPSS ≥ 8 gradually increased with age. For UUI, the prevalence also increased with age in ≥ 40 years age group. In the 20–29 and 30–39 years groups, the prevalence of UUI was higher in comparison to the 40–49 years group (19.6% in 20–29 years group, 18.6% in the 30–39 years group, and 14.3% in the 40–49 years group) (Fig. 5). Ex- and current-smoker showed a higher prevalence of OAB, UUI, nocturia, and IPSS ≥ 8, especially in the young age groups (Figs. 4, 5, 6 and 7).

The impact of the smoking status on OAB in each age group is shown in Fig. 4. When the risk was set at 1 for non-smokers, the risk ratios for ex-smokers and current smokers were 4.0 and 2.6, respectively, at 20–29 years, 1.7 and 1.7 at 30–39 years, 1.2 and 1.5 at 40–49 years, 1.2 and 1.5 at 50–59 years, 1.1 and 1.2 at 60–69 years, and 1.0 and 1.3 at ≥ 70 years. A smoking habit had a stronger influence on younger participants (i.e., the 20–39 years age group) than it did on individuals ≥ 60 years of age. The impact of the smoking status on UUI in each age group is shown in Fig. 5. When the risk was set as 1 for non-smokers, the risk ratios for ex- and current smokers were 3.9 and 2.2, respectively, at 20–29 years, 1.8 and 1.8 at 30–39 years, 1.3 and 1.4 at 40–49 years, 1.3 and 1.3 at 50–59 years, 1.1 and 1.1 at 60–69 years, and 1.1 and 1.3 at ≥ 70 years. A smoking habit had a stronger influence on younger participants than it did on individuals ≥ 60 years of age. The impact of smoking status on nocturia in each age group is shown in Fig. 6. When the risk was set as 1 for non-smokers, the risk ratios for ex- and current smokers were 2.2 and 1.6, respectively, at 20–29 years, 1.5 and 1.2 at 30–39 years, 1.2 and 1.1 at 40–49 years, 1.1 and 1.0 at 50–59 years, 1.1 and 1.0 at 60–69 years, and 1.1 and 1.0 at ≥ 70 years. A smoking habit had a stronger influence on younger participants than it did on individuals of ≥ 60 years of age. The impact of smoking status on IPSS ≥ 8 in each age group is shown in Fig. 7. When the risk was set as 1 for non-smokers, the risk ratios for ex- and current smokers were 3.5 and 2.5, respectively at 20–29 years, 1.9 and 1.4 at 30–39 years, 1.4 and 1.6 at 40–49 years, 1.2 and 1.3 at 50–59 years, 1.2 and 1.2 at 60–69 years, and 1.2 and 1.3 at ≥ 70 years. A smoking habit had a stronger influence on younger participants than it did on individuals of ≥ 60 years of age. These trends were also observed in UUI (ICIQ-SF definition) and nocturia (IPSS definition) (Supplementary Figs. S3 and S4). Smoking also had a greater influence on the risk of UUI and nocturia in the younger age group.

**Figure 8:**
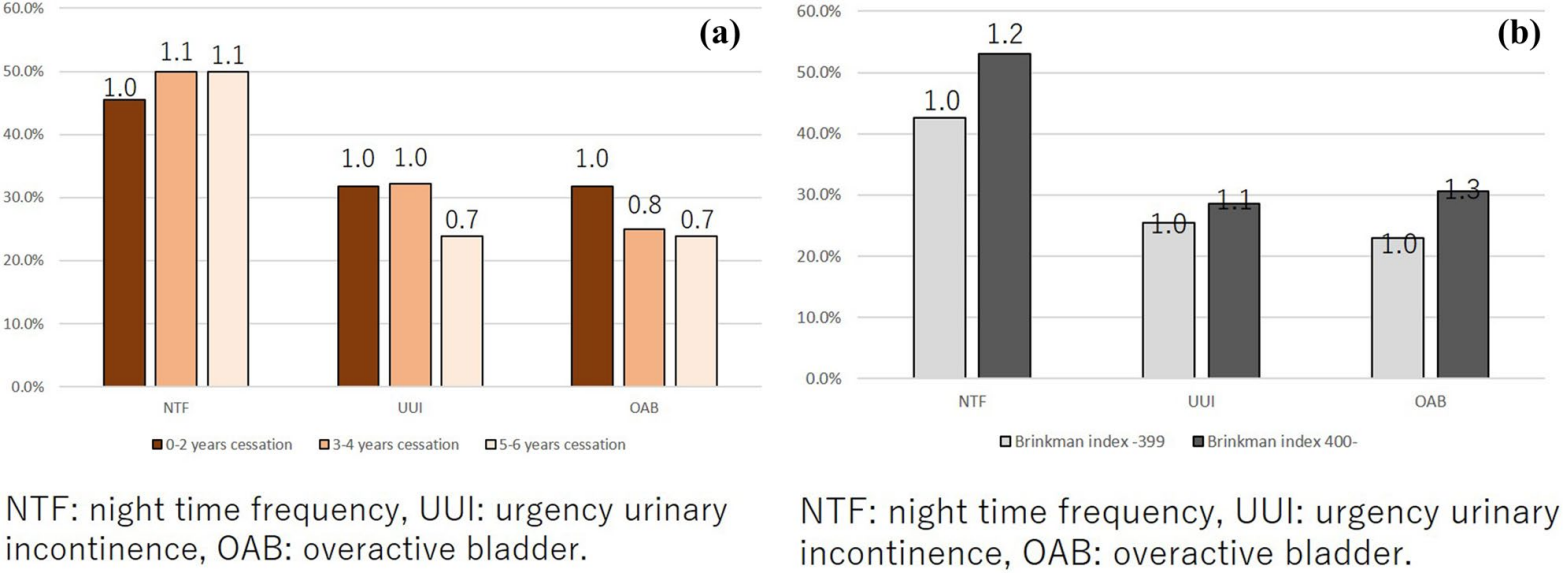
(**a**) The prevalence of nocturia, UUI, and OAB according to the duration of smoking cessation; and (**b**) the prevalence of nocturia, UUI, and OAB according to the Brinkman index.

The prevalence risk was higher in the relatively young age group (20–39 years) in comparison to the elderly participants; thus, we selected the young age group (20–39 years) to compare the detailed smoking status. For ex-smokers, the longer smoking cessation group (5–6 years smoking cessation) showed a lower prevalence of UUI and OAB in comparison to the 0–2 years cessation group (0.7 and 0.7, respectively, at 5–6 years cessation; 1.0 and 0.8, at 3–4 years cessation). In contrast, the prevalence of nocturia did not differ according to the duration of smoking cessation (1.1 at 5–6 years cessation; 1.1 at 3–4 years cessation) (Fig. 8a,b). Based on these data, a longer period of smoking cessation might reduce the symptoms of UUI and OAB in the younger age group. Among current-smokers, the higher Brinkman index (BI) group (BI ≥ 400) showed a higher prevalence of nocturia, UUI, and OAB than the lower BI (BI < 400) group (relative risk vs. BI < 400 group: 1.2 for nocturia, 1.1 for UUI, and 1.3 for OAB).

## Discussion

We performed this large population-based cross-sectional study to reveal the impact of a smoking habit on male LUTS in a total of 9042 male participants. Previous large cross-sectional cohort studies include the EpiLUTS study, with a total of 30,000 participants (12,139 males) and the EPIC study, with a total of 19,165 participants (approximately 7500 males)^2,19^. In comparison to these previous studies, the present study included a large number of Asian-Japanese males. We firstly screened by age in Japanese population distribution, then asked smoking status. This study included 3545 (39.2%) non-smokers, 3060 (33.8%) ex-smokers, and 2437 (27.0%) current-smokers, as well as detailed information about smoking, including the duration of smoking cessation, smoking history, and the volume of daily smoking. We revealed that current- and ex-smokers had worse urinary symptoms based on an established questionnaire using the OABSS, ICIQ-SF, and IPSS to evaluate urinary symptoms^13^^17^.

This study also confirmed that the prevalence of OAB, storage symptoms, and nocturia were positively correlated with age. Although the prevalence of UUI gradually increasing with age after 40 years of age, a certain number of men in the young age group had UUI (19.6% in the 20–29 years group and 18.6% in 30–39 years group vs. 14.3% in 40–49 years group). Homma et al. examined a total of 2100 Asian-Japanese men to assess OAB symptoms in 2005^5^. In that study, OAB was seen in 14% men, while the incidence in the current study was 20.3%, and the tendency according to age was almost the same as in the present study.

The differences of LUTS between non-smokers and current- or ex-smokers were more pronounced in the young age groups than in the elderly age group (Figs. 4, 5, 6 and 7). However, the differences diminished with age. This phenomenon was observed not only for UUI and OAB but also for daytime frequency, nocturia, and storage symptoms. Wang et al. revealed that cigarette smoking increased the risk of developing LUTS in both sexes based on 4723 individuals, including 1551 men with a risk ratio of 1.32 for storage symptoms, 1.50 for voiding symptoms, and 1701 for postmicturition symptoms^20^. In that study, the mean age ± SD of the male participants was 39.7 ± 14.6 years. The cohort of the present study was relatively young in comparison to the other cross-sectional studies. Booth et al. investigated a total of 503 males of ≥ 60 years of age (mean age: ≥ 65 years of age) and revealed no differences in the IPSS scores of smokers and non-smokers^21^. Furukawa et al. examined 817 patients, including 514 males with type 2 diabetes mellitus and showed that no differences in mild or moderate nocturia and an inverse association with nocturia with a prevalence ratio of 0.47^22^. The mean ± SD age of the overall cohort was 61.9 ± 11.1 years. Regarding urgency, the prevalence of urgency in older current or former-smoker was higher than that in non-smokers^23^. In a study of former smokers, the risk ratio was 1.14 (13.3% in non-smokers vs. 15.1% in former smokers) at 75–85 years but 1.97 (3.9% in non-smokers vs. 7.7% in former smokers) at 60–74 years^24^. These phenomena were also observed in night time frequency, in age 50 or older there were no differences in terms of night time frequency between each smoking status. Based on these previous studies and the current study, cigarette smoking was associated with a high risk of developing male LUTS at a relatively young age, but the differences in the cigarette smoking status were diminished because other risk factors for LUTS, including age, diabetes mellitus, hypertension, and cardiovascular disease increased with age^9,25–30^. The current study showed positive association between smoking and LUTS in all ages; however, the risk ratio differed in each age group. Most studies evaluated the impact of smoking on LUTS in an overall cohort; however, LUTS are highly affected by aging; thus, the impact of smoking should be assessed in each age group^23^. This study showed that in age group 20–29 ES group showed higher prevalence of OAB, UUI, nighttime frequency, and IPSS 8 or more than CS group. We speculate that in Japanese law cigarette smoking are permitted 20 or more years old, thus ES does not show shorter cigarette smoking status than CS comparing to the ageing group 30 or more years old.

We revealed that current- and ex-smokers showed a higher prevalence of day-time frequency, nocturia, UUI, OAB, PMD, and IPSS ≥ 8. Previous studies have shown that the correlation between cigarette smoking and LUTS is still controversial^11^. Smoking has also been shown to impair endothelial NOS-mediated vascular dilation in young men^1^. While the detailed mechanism is still unknown, smoking-induced atherosclerosis is thought to be a pathway influencing the development of LUTS^31^. Regarding the vascular damage associated with cigarette smoking, some data have suggested that it may lower testosterone levels^1,32–34^. Arterial occlusive disease may lead to chronic bladder ischemia, bladder hyperactivity, and morphologic bladder wall changes^31^. Aging usually reduces the bladder blood flow and causes vascular endothelial dysfunction, resulting in atherosclerosis and hypertension^35,36^. Smoking may also play a role in the pathogenesis of pelvic flow dysfunction and urinary incontinence^37^. This effect might be considered to reflect the reported relationship between smoking and LUTS.

Previous reports showed that smoking caused endothelial sclerosis and a worse bladder function. Recent studies showed that phosphodiesterase type 5 (PDE-5) inhibitor improved endothelial sclerosis^38^. Tadalafil ,a PDE-5 inhibitor, inhibits PDE-5 which is distributed in the vascular endothelium and smooth muscles of the bladder, and also increases the concentration of cyclic guanosine monophosphate produced in the response to the level of nitric oxide in tissue^39^. Based on these mechanisms, the blood flow and oxygen supply increased in the tissue and resulted in the improvement of the blood flow of the lower urinary tract^39^. Gacci et al. reported a meta-analysis on the use of PDE-5 inhibitors in the management of LUTS due to BPH and revealed that PDE-5 inhibitors were especially beneficial in younger age patients^40^. According to these results, for relatively young LUTS patients, endothelial sclerosis had a greater impact on LUTS in comparison to elderly patients with a high prevalence of BPH or other age-related factors. PDE-5 inhibitor treatment might be beneficial for young current- or ex-smokers with LUTS.

The present study was associated with several limitations. First, the survey lacked detailed health information, such as complicating diseases and medication use. A certain number of individuals who showed high symptom scores would have been receiving medication for LUTS. Like other cross-sectional cohort studies, the present study could not obtain other risk factors for LUTS. Second, we could not reveal the mechanism underlying the impact of smoking habits on LUTS. In current- and ex-smokers, almost all of the questionnaire scores were higher and the prevalence of almost every types of LUTS were higher in comparison to non-smokers. The prevalence of SUI and MUS was higher among current-smokers than among ex-smokers. However, the prevalence rates of other LUTS in ex-smokers were higher or almost the same as those in current-smokers. Though detailed mechanisms how smoking affects LUTS have not been established, the possible mechanisms through which smoking affects LUTS include arteriosclerosis or impaired endothelial vascular dilation. Although this study did not show that the influence came from arteriosclerosis, the results of the differences in symptoms according to smoking habits support the hypothesis that smoking might cause arteriosclerosis in the bladder and seems to result in urinary symptoms. These phenomena were highly observed in young cases because the other risk factors were low at young age. The third limitation is a selection bias. This study used a web-based internet survey, which was completed using a smart phone, tablet or PC. The EPIC study in 2005 used computer-assisted telephone interviews and the EpiLUTS study in 2006–2008 used an Internet survey after an e-mail invitation^2,19^. In comparison to other studies, due to the widespread of Internet access during these 15 years, more people were familiar with web-based surveys. In the EPIC study, only 19,154 of 58,139 respondents (33.9%) agreed to complete the LUTS questionnaire; thus, the authors suggested that the bias was a limitation of their study. In the current study 9042 of 10,000 (90.4%) respondents agreed to complete the additional LUTS survey after an initial check of their smoking status. This high acceptance rate was come from the participants who were registered as monitoring panel of research company.

In conclusion, this was the first large cross-sectional population-based study to reveal the impact of cigarette smoking on male LUTS. Current- and ex-smokers showed a higher prevalence of male LUTS. This phenomenon was more pronounced in the young age group (20–29).

## Supplementary information

Supplementary Information

Supplementary Information

Supplementary Information

Supplementary Information

Supplementary Information

Supplementary Information

## Data Availability

The raw data used to create the tables and figures are available as a supplementary file.
