# Supplementary material for: The impact of smoking on male lower urinary tract symptoms (LUTS)
Source: Sci Rep. 2020 Nov 19;10:20212. doi: 10.1038/s41598-020-77223-7 (PMC7678847; doi:10.1038/s41598-020-77223-7)
Supplement: Supplementary file 1 — Supplementary Information [file 41598_2020_77223_MOESM1_ESM.docx]

TITLE:

The impact of smoking on male lower urinary tract symptoms (LUTS)

Authors:

Takashi Kawahara, Hiroki Ito, Hiroji Uemura

| Suppl Table 1 |  |  |  |  |
| --- | --- | --- | --- | --- |
| Prefecture | All | Non-Smoker | Ex-Smoker | Current-Smoker |
| Aichi | 572 | 168 | 167 | 237 |
| Ehime | 75 | 20 | 22 | 33 |
| Ibaraki | 155 | 31 | 55 | 69 |
| Okayama | 114 | 40 | 34 | 40 |
| Okinawa | 37 | 11 | 8 | 18 |
| Iwate | 61 | 15 | 19 | 27 |
| Gifu | 136 | 30 | 52 | 54 |
| Miyazaki | 35 | 9 | 9 | 17 |
| Miyagi | 145 | 38 | 60 | 47 |
| Kyoto | 192 | 43 | 67 | 82 |
| Kumamoto | 73 | 19 | 30 | 24 |
| Gunma | 83 | 13 | 39 | 31 |
| Hiroshima | 184 | 45 | 70 | 69 |
| Kagawa | 60 | 13 | 26 | 21 |
| Kochi | 31 | 15 | 5 | 11 |
| Saga | 46 | 12 | 16 | 18 |
| Saitama | 598 | 160 | 196 | 242 |
| Mie | 105 | 24 | 34 | 47 |
| Yamagata | 51 | 17 | 13 | 21 |
| Yamaguchi | 93 | 21 | 34 | 38 |
| Yamanashi | 36 | 14 | 10 | 12 |
| Shiga | 91 | 26 | 31 | 34 |
| Kagoshima | 58 | 22 | 18 | 18 |
| Akita | 40 | 14 | 9 | 17 |
| Niigata | 113 | 32 | 36 | 45 |
| Kanagawa | 929 | 231 | 334 | 364 |
| Aomori | 68 | 19 | 29 | 20 |
| Shizuoka | 185 | 43 | 71 | 71 |
| Ishikawa | 72 | 13 | 27 | 32 |
| Chiba | 535 | 135 | 194 | 206 |
| Osaka | 776 | 205 | 249 | 322 |
| Oita | 42 | 11 | 17 | 14 |
| Nagasaki | 63 | 24 | 15 | 24 |
| Nagano | 112 | 36 | 36 | 40 |
| Tottori | 21 | 8 | 4 | 9 |
| Shimane | 31 | 7 | 10 | 14 |
| Tokyo | 1303 | 369 | 416 | 518 |
| Tokushima | 57 | 10 | 20 | 27 |
| Tochigi | 107 | 33 | 32 | 42 |
| Nara | 132 | 30 | 50 | 52 |
| Toyama | 66 | 15 | 17 | 34 |
| Fukui | 37 | 15 | 12 | 10 |
| Fukuoka | 254 | 74 | 92 | 88 |
| Fukushima | 89 | 25 | 29 | 35 |
| Hyogo | 517 | 136 | 172 | 209 |
| Hokkaido | 418 | 139 | 156 | 123 |
| Wakayama | 44 | 7 | 18 | 19 |
|  |  |  |  |  |
