# Supplementary figures and images for: The impact of smoking on male lower urinary tract symptoms (LUTS)
Source: Sci Rep. 2020 Nov 19;10:20212. doi: 10.1038/s41598-020-77223-7 (PMC7678847; doi:10.1038/s41598-020-77223-7)

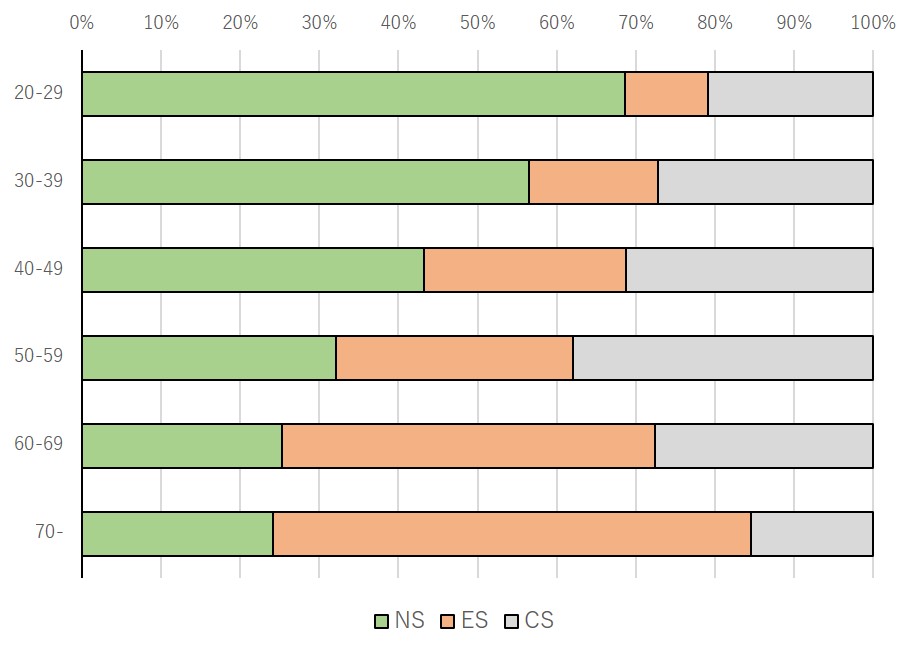

Supplement: Supplementary file 2 — Supplementary Information [file 41598_2020_77223_MOESM2_ESM.jpg]

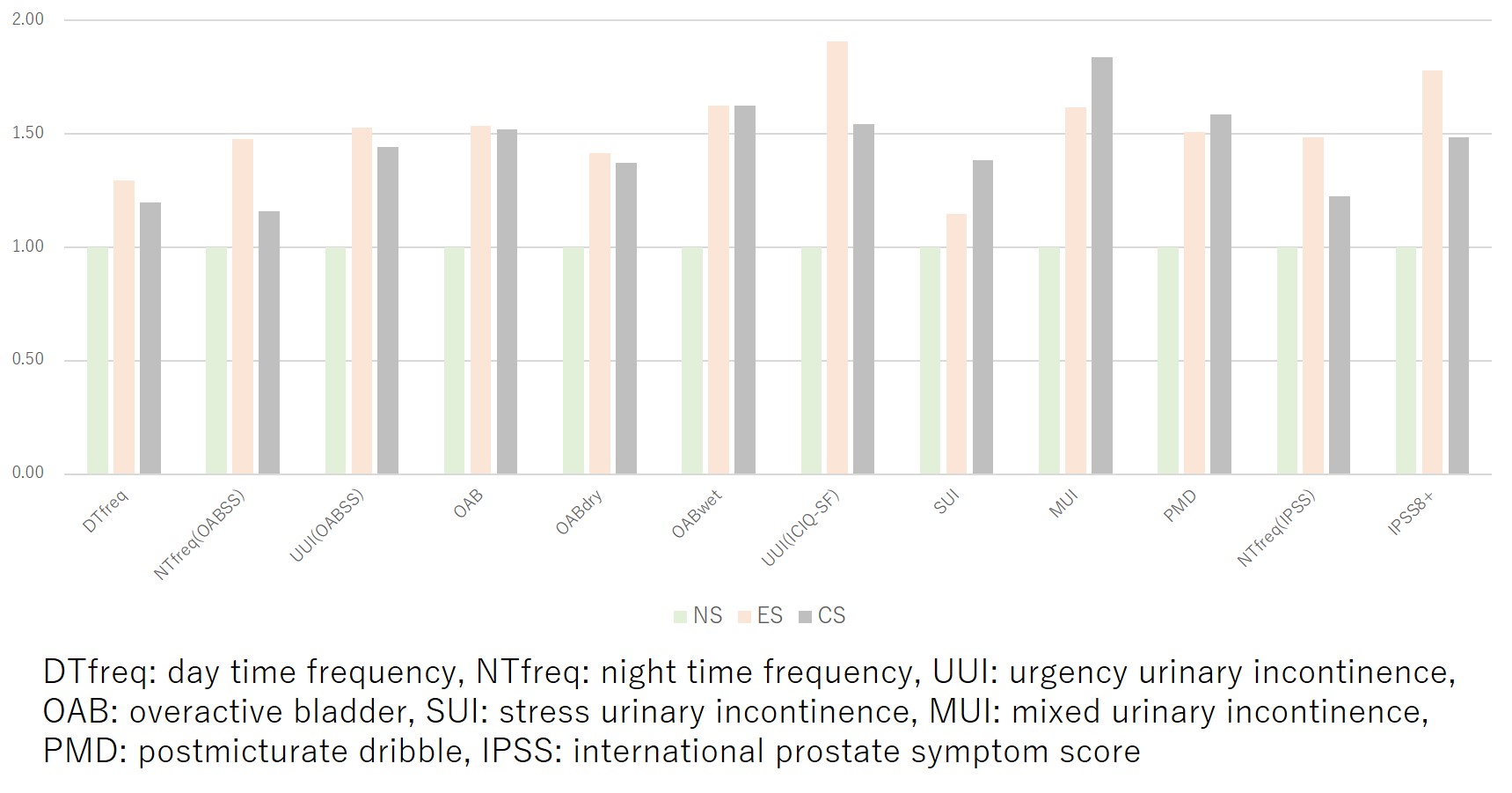

Supplement: Supplementary file 3 — Supplementary Information [file 41598_2020_77223_MOESM3_ESM.jpg]

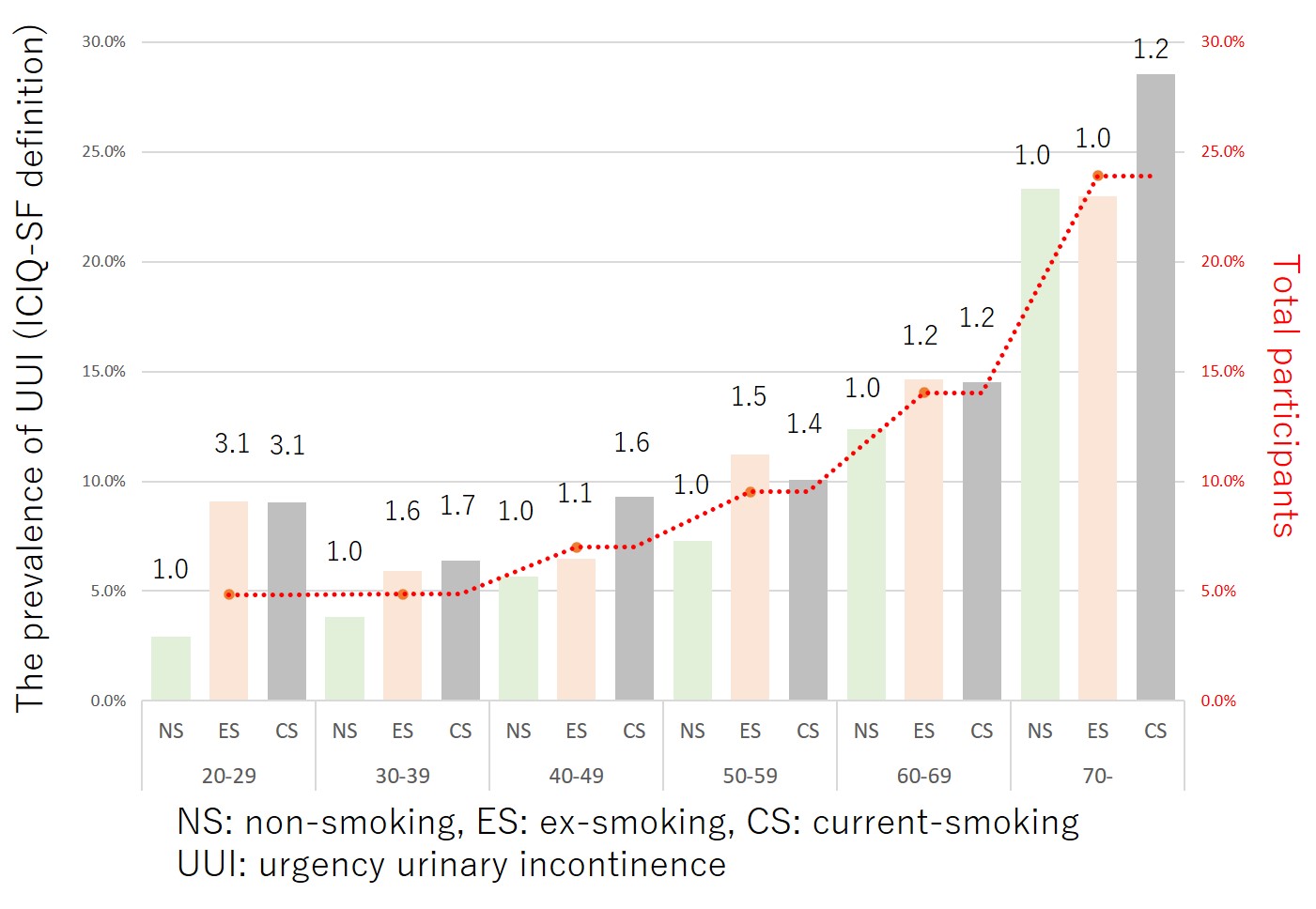

Supplement: Supplementary file 4 — Supplementary Information [file 41598_2020_77223_MOESM4_ESM.jpg]

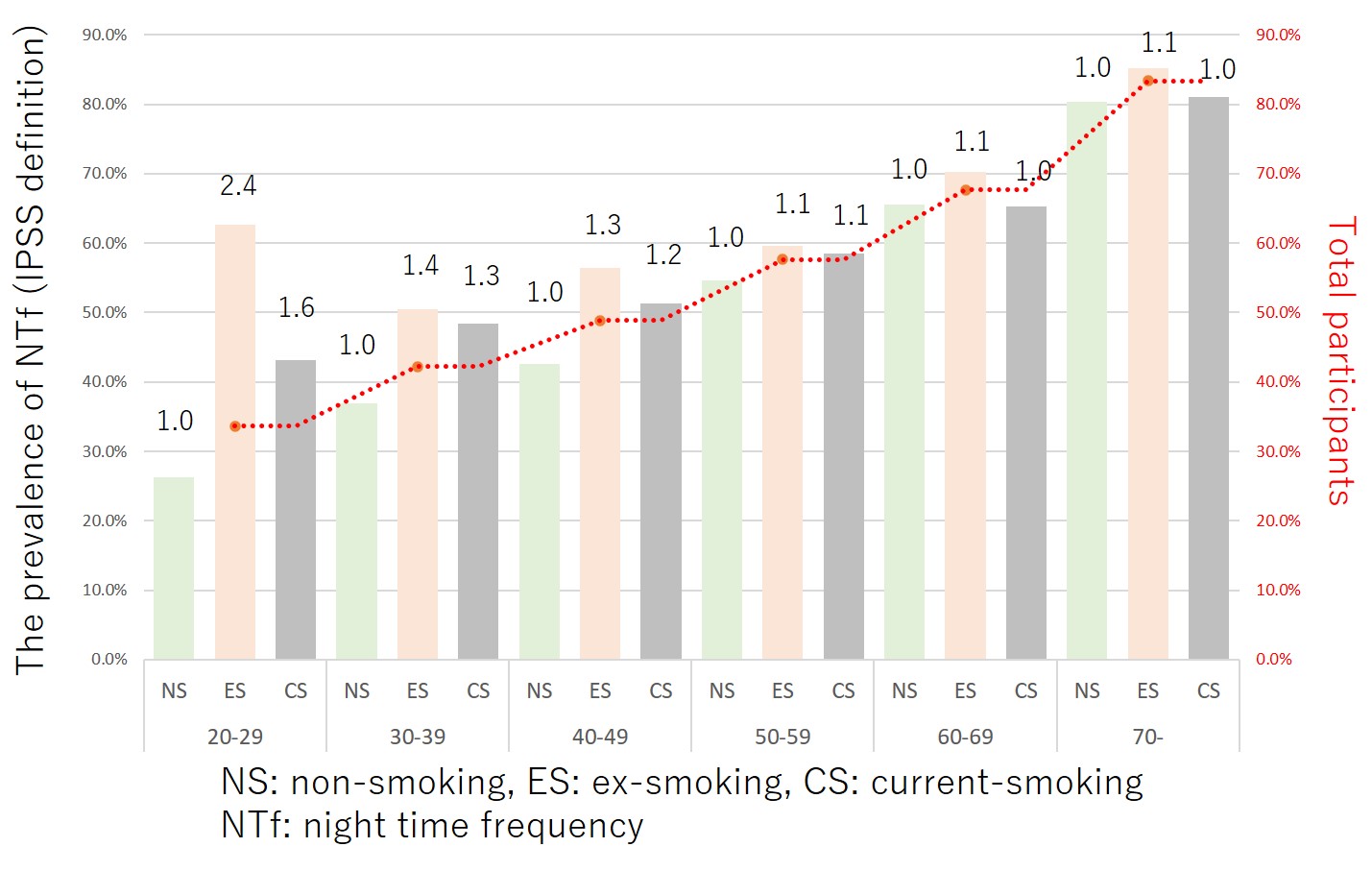

Supplement: Supplementary file 5 — Supplementary Information [file 41598_2020_77223_MOESM5_ESM.jpg]
