# Supplementary material for: The impact of smoking on male lower urinary tract symptoms (LUTS)
Source: Sci Rep. 2020 Nov 19;10:20212. doi: 10.1038/s41598-020-77223-7 (PMC7678847; doi:10.1038/s41598-020-77223-7)
Supplement: Supplementary file 6 — Supplementary Information [file 41598_2020_77223_MOESM6_ESM.docx]

**Supplementary Materials:**

**Supple Table 1** Regional distribution of the participants

**Supple Figure 1** Number of targeted screening participants and ratio of smoking habits in each generation.

**Supple Figure 2** The difference in the relative risk for day-time frequency, nocturia (OABSS), UUI (OABSS definition), OAB, OAB dry, OAB wet, UUI (ICIQ-SF), SUI, MUI, PMD, nocturia (IPSS definition), and IPSS≥8 in non-, ex-, and current-smokers.

**Supple Figure 3** The prevalence of UUI (ICIQ-definition) in each age group among the different smoking habit groups.

**Supple Figure 4** The prevalence of noctruia (IPSS definition) in each age group among the different smoking habit groups.
